# Influence of extent and age at corpus callosotomy on seizure outcomes. A single center experience

**DOI:** 10.1002/epi4.12819

**Published:** 2023-10-18

**Authors:** Nitish Chourasia, Scellig S. D. Stone, Melissa Tsuboyama, Joseph R. Madsen, Morgan Ryan, Bo Zhang, Mark H. Libenson, Jeffrey Bolton, Chellamani Harini

**Affiliations:** ^1^ Division of Epilepsy and Clinical Neurophysiology, Department of Neurology Boston Children's Hospital Massachusetts Boston USA; ^2^ Department of Neurology Boston Children’s Hospital Boston Massachusetts USA; ^3^ Biostatistics and Research Design Center Institutional Centers for Clinical and Translational Research, Boston Children’s Hospital Boston Massachusetts USA; ^4^ Present address: Le Bonheur Neuroscience Institute, Le Bonheur Children's Hospital Tennessee Memphis USA

**Keywords:** age at surgery, corpus callosotomy, epilepsy surgery, pediatric epilepsy, refractory epilepsy

## Abstract

Corpus callosotomy (CC) is a palliative treatment for drop seizures in patients with drug‐resistant nonlocalizable epilepsy. We compared drop seizure outcomes between patients undergoing anterior CC versus complete CC and examined factors impacting outcomes for drop seizures including age at CC and duration of epilepsy. A retrospective review of patients who underwent CC between 2003 and 2022 with a minimum of 6 months postsurgical follow‐up was included. Outcome measure for drop seizures included seizure reduction ≥50% from baseline as well as elimination of drop seizures. Thirty‐eight patients were included. Overall, ≥50% reduction in drop seizures occurred in nearly 70% (23 out of 33) patients with complete elimination in 58% (19 out of 33). Compared with anterior CC (*n* = 13), patients undergoing complete CC (*n* = 25) had increased likelihood of ≥50% reduction (*p* = 0.006) or elimination (*p* = 0.024) of drop seizures. Regression analysis showed that complete CC was the primary predictor for improved drop seizure outcomes (elimination, *p* = 0.014 or ≥50% reduction, *p* = 0.006), while age at CC and duration of epilepsy did not impact the outcomes. Compared to anterior CC, complete CC was significantly more likely to lead to improvement/freedom from drop seizures. Age at CC or duration of epilepsy did not influence drop seizure outcomes.

## 1 INTRODUCTION

Corpus callosotomy (CC) is a palliative procedure performed in patients with drug‐resistant generalized (excluding genetic generalized epilepsy) or multifocal epilepsy, especially for treating drop seizures.^1^, ^2^, ^3^ Surgical sectioning of the corpus callosum may commonly include anterior two‐thirds (anterior CC) or the entire corpus callosum (complete CC) or rarely the posterior callosum alone (posterior CC). The significance of the extent of CC on seizure outcomes has been debated. A recent meta‐analysis and a systematic review suggested that seizure freedom rates were lower with anterior two‐thirds CC compared to complete CC.^2^, ^3^ However, this inference of the systematic review was based on patients undergoing anterior CC only^4^ and a study that failed to demonstrate a difference in seizure outcomes based on extent of disconnection.^5^ Thus, clarifying the impact of the extent of callosal disconnection on seizure outcomes could benefit from additional data.

The influence of age at CC on seizure outcomes has not been established. Evaluating the impact of age at CC on seizure outcomes from published data is challenging due to varying outcome definitions in relatively older cohorts undergoing CC (late first decade or second decade).^2^ Overall, children were reported to have better seizure outcomes when compared to adults.^2^, ^6^, ^7^ Recent reports indicate that CC was associated with excellent seizure outcomes (freedom from or >80% reduction of epileptic spasms) in ~50% of patients with West syndrome (mean age at CC was 22.6 months).^8^, ^9^ This could imply that either age at CC or seizure type (epileptic spasms) could potentially influence seizure outcomes following CC.

We conducted a single‐center retrospective study to (a) compare seizure outcomes for drop seizures between anterior two‐thirds versus complete CC, (b) evaluate the impact of age at CC surgery on drop seizure outcomes.

## 2 METHODS

The prospectively maintained epilepsy surgery database at Boston Children's Hospital (BCH) was used to identify all patients who underwent CC between 2003 and 2022. Patients who had CC at our center with a minimum of 6 months postoperative follow‐up were included. Patients who had other procedures such as Vagus Nerve Stimulator (VNS) implantation were included in the study. Patients who underwent staged CC (ie, anterior two‐thirds CC followed by complete CC) were excluded due to the inability to attribute seizure outcomes to a particular procedure when sequential procedures were performed. For example, for patients who may have poor outcomes following sequential 2‐stage completion of CC, assigning these results to either surgery would inaccurately represent procedural outcomes. Drop seizures included falls secondary to atonic, tonic or myoclonic seizures.

### 2.1 Outcome measures

Primary outcome for drop seizures was defined as (a) reduction in drop seizures by ≥50% (provided residual drop seizures in these patients were noninjurious), (b) complete freedom drop seizures. Seizure frequency pre‐ and post‐CC (at last follow‐up) were also quantified for other seizure types.

At our center, patients with refractory epilepsy being assessed for a possible CC underwent a presurgical work‐up that included brain MRI, electroencephalogram (EEG) recordings (ictal and interictal). Seizure type and frequency were based on clinical documentation obtained from parental recall at clinic visits. Seizure type was confirmed by video‐EEG (vEEG) monitoring in most patients pre‐CC and in some cases post‐CC. Patients with nonlocalizing ictal EEG who were not deemed to have a resectable epileptogenic focus on MRI were offered CC. All decisions were based on the consensus derived at a multidisciplinary epilepsy surgery conference. In general, complete CC was performed in patients with moderate to severe cognitive impairment. Cognitive delay was an important but not the sole determinant of the extent of CC. Decisions regarding the extent of CC were individualized for each patient. Caregiver views regarding the severity of epilepsy, patient's functional status as well as concerns regarding potential complications from complete disconnection were also incorporated before deciding on the extent of CC. Extent of disconnection was guided by intraoperative anatomic landmarks with neuro‐navigation, confirmed in most by intraoperative or postoperative brain MRI.

This study was approved by the BCH Institutional Review Board.

### 2.2 Statistical analysis

Mean and standard deviation were used for continuous variables and counts with percentages were used for categorical variables for summary statistics. The demographic and clinical characteristics as well as the seizure outcomes (drop seizures) associated with the extent of CC were compared. Student's t‐test was conducted for continuous variables and Fisher's exact test for categorical variables. We examined the impact of age at CC, duration of epilepsy and other clinical and demographic variable on drop seizure outcomes (≥50% reduction drop seizures and complete freedom drop seizures) using univariate and multivariate logistic regression analysis. Age at surgery and other variables with a *p*‐Value < 0.2 in univariate logistic regression were included in the multivariate logistic regression. Statistical significance was defined as *p* < 0.05. Data analysis was performed using R version 4.2.0.

## 3 RESULTS

Among the 44 CC patients identified from the surgical database, six were excluded (three due to multistage CC and three due to insufficient follow up at our institution post‐CC). A total of 38 patients (71% males) were included, 13 who underwent anterior two‐thirds CC and 25 who underwent complete CC. All patients underwent open craniotomy except one patient who had laser interstitial thermal therapy (LITT) callosotomy. Thirteen patients had VNS placement before any CC (anterior or complete) while two had VNS placement after CC.

### 3.1 Clinical characteristics for the overall group

Mean age at seizure onset was 2.2 ± 2.7 years. The mean duration of epilepsy was 8.7 ± 6.0 years and mean duration of follow up after CC was 45.7 ± 33 months. The mean age at surgery was 11.1 ± 6.3 years. The mean number of antiseizure medications presurgery was 3.2 ± 0.8 and postsurgery was 3.0 ± 0.9. Moderate to severe cognitive impairment was noted in 33 out of 38 patients (87%). Etiology was known in 21 patients (55%).

More than one seizure type was noted in 33 of the 38 patients (87%). Seizure types included varying combinations of tonic (*n* = 24), atonic (*n* = 6), generalized tonic clonic (GTC) (*n* = 10), atypical absence (*n* = 8), myoclonic (*n* = 5), focal with impaired awareness (*n* = 2) and epileptic spasms (*n* = 2). Multiple daily drop seizures were noted in 33 of the 38 patients. Based on vEEG, drop seizures were due to tonic (*n* = 22), atonic (*n* = 6), myoclonic (*n* = 1) seizures. Drop seizures type was undetermined in four patients as the drop seizures were not captured on video EEG and the drop seizure type (atonic vs tonic) could not be determined based on the caregiver description.

MRI demonstrated congenital abnormalities in 23 patients, including periventricular nodular heterotopia (*n* = 2), bilateral polymicrogyria (*n* = 5), lissencephaly (*n* = 1), focal cortical dysplasia (*n* = 2), and nonspecific white matter abnormalities (*n* = 6). Acquired abnormalities were seen in 7 patients and included strokes (*n* = 6) and traumatic brain injury (*n* = 1). The extent of CC was confirmed by intraoperative and/or postoperative brain MRI in 34 of 38 patients (90%).

### 3.2 Complications

The median length of hospital stay (LOS) for the entire cohort was 5 days (IQR 4–7.75). The reasons for LOS >5 days were depressed mental status (*n* = 5), poor oral intake (*n* = 6), delayed ambulation (*n* = 3), transient hemiparesis (*n* = 1), bilateral intraventricular hemorrhage needing ventriculo‐peritoneal shunt placement (*n* = 1) and pneumonia (*n* = 2). Disconnection syndrome was not noted in any patient.

### 3.3 Outcome data

#### 3.3.1 Drop attacks

At the last follow‐up, following CC (anterior and complete), nearly 70% patients (23 of the 33 patients) had ≥50% reduction in drop seizures, with complete elimination of drop seizures in 19 of these 23 patients. In the other four patients, residual drop seizures were not associated with injuries and represented a change from the preoperative status. Freedom from drop seizures occurred in 3 of 11 (27%) with anterior two‐thirds CC vs 16 of 22 (73%) with complete CC (*p* = 0.024).

#### 3.3.2 Other seizures

Among the other seizure types, ≥50% reduction in seizures was noted in five of the eight patient (63%) with atypical absence, 6 of the 10 (60%) with GTC.

### 3.4 Comparison between anterior two‐thirds vs complete CC

Table 1 details the comparison of demographic/clinical characteristics and seizure outcomes between patients following anterior two‐thirds vs complete CC. Most of the demographic/clinical variables did not significantly differ between the groups (*p* > 0.05). Number of seizure types postsurgery differed between the groups (*p* = 0.012). Complete CC was significantly associated with ≥50% reduction drop seizures when compared to anterior two thirds CC (86.4 vs 36.4%, *p* = 0.006). Similarly, complete CC was significantly associated with elimination of drop seizures when compared to anterior two‐thirds CC (72.7% vs 27.3%, *p* = 0.024).

**TABLE 1:** Comparison of demographic and clinical characteristics between anterior two‐thirds corpus callosotomy CC (*n* = 13) and complete CC (*n* = 25).

Characteristics [mean (SD)] or *n* (%)	Anterior two‐thirds Callosotomy	Complete Callosotomy	*p*‐Value
	(*n* = 13)	(*n* = 25)	
Age at seizure onset (years)	3.32 (2.75)	1.58 (2.59)	0.062
Sex			
Female	6 (46.2%)	5 (20.0%)	0.135
Male	7 (53.8%)	20 (80.0%)	
Duration of Epilepsy Prior to surgery (years)	8.14 (5.40)	8.97 (6.39)	0.691
Age at surgery (years)	11.46 (6.67)	10.92 (6.16)	0.804
Duration of Follow up (years)	46.31 (37.70)	45.44 (32.27)	0.941
MRI abnormality			
Yes	6 (46.2%)	17 (68.0%)	0.295
No	7 (53.8%)	8 (32.0%)	
Etiology known			
Yes	5 (38.5%)	16 (64.0%)	0.178
No	8 (61.5%)	9 (36.0%)	
Duration of hospital stay after surgery (days)	6.00 (2.74)	7.28 (5.47)	0.435
No. of antiseizure medications presurgery	3.23 (0.83)	3.20 (0.82)	0.913
No. of antiseizure medications postsurgery	3.23 (0.83)	2.80 (0.96)	0.178
No. of seizure types presurgery	2.31 (0.63)	2.20 (0.76)	0.665
No. of seizure types postsurgery	1.92 (0.64)	1.28 (0.74)	0.012
Interictal EEG with lateralizing features			
Yes	2 (15.4%)	7 (28.0%)	0.456
No	11 (84.6%)	18 (72.0%)	
≥50% Reduction in drop seizures	*N* = 11	*N* = 22	0.006
Yes	4 (36.4%)	19 (86.4%)	
No	7 (63.6%)	3 (13.6%)	
Complete Freedom from drop seizures	*N* = 11	*N* = 22	0.024
Yes	3 (27.3%)	16 (72.7%)	
No	8 (72.7%)	6 (27.3%)	

Abbreviations: ASM, antiseizure medications; EEG, electroencephalogram; F, female; Y, years.

### 3.5 Determinants of drop seizure outcome

Table 2 details logistic regression analysis for seizure outcome ≥50% reduction in drop seizures Multivariate regression analysis showed that complete CC (Odds Ratio (OR) 11.32, confidence interval‐CI (2.18, 76.75), *p* = 0.006) was the only factor that was significantly associated with ≥50% reduction in drop seizures. Age at surgery (OR 1.02, CI‐[0.89, 1.18], *p* = 0.752) did not affect the seizure outcomes. Duration of epilepsy, sex, presence of known etiology, abnormal MRI, presurgery variables did not affect the probability of obtaining ≥50% reduction in drop seizures.

**TABLE 2:** Univariable and multivariable logistic regression for the probability of >50% reduction drop seizure (^a^ *N* = 33).

	OR (95% CI)	*p*‐Value
Univariable logistic regression		
Age at surgery	1.01 (0.90, 1.14)	0.875
Duration of epilepsy prior to surgery (years)	1.03 (0.91, 1.19)	0.634
Age at seizure onset	0.87 (0.66, 1.13)	0.300
Female	0.82 (0.16, 4.76)	0.817
Etiology known	1.88 (0.41, 8.79)	0.414
MRI abnormality	1.52 (0.31, 7.21)	0.593
Interictal EEG with lateralizing features	1.41 (0.25, 11.11)	0.709
No. of seizure types presurgery	1.26 (0.44, 3.85)	0.666
No. of antiseizure medications presurgery	0.90 (0.32, 2.55)	0.839
Complete callosotomy	11.08 (2.16, 73.36)	0.006
Multivariable Logistic Regression		
Age at surgery	1.02 (0.89, 1.18)	0.752
Complete callosotomy	11.32 (2.18, 76.75)	0.006

^a^ *N* = 33 (33 out of 38 had drop seizures).

Table 3 details logistic regression analysis for complete freedom from drop seizures.

**TABLE 3:** Univariable and multivariable logistic regression for the probability of complete freedom drop seizure (^a^ *N* = 33).

	Odds ratio (95% confidence interval)	*p*‐Value
Univariable logistic regression		
Age at surgery	0.95 (0.84, 1.06)	0.356
Duration of epilepsy prior to surgery (years)	0.97 (0.86, 1.09)	0.642
Age at seizure onset	0.95 (0.72, 1.22)	0.658
Female	0.89 (0.19, 4.41)	0.886
Etiology known	1.29 (0.31, 5.37)	0.727
MRI abnormality	1.20 (0.27, 5.26)	0.803
Interictal EEG with lateralizing features	2.77 (0.52, 21.49)	0.263
No. of seizure types presurgery	0.90 (0.33, 2.39)	0.823
No. of antiseizure medications presurgery	0.47 (0.15, 1.23)	0.146
Complete callosotomy	7.11 (1.52, 42.0)	0.018
Multivariable logistic regression		
Age at surgery	0.96 (0.84, 1.10)	0.558
No. of antiseizure medications presurgery	0.38 (0.10, 1.14)	0.107
Complete callosotomy	9.73 (1.81, 74.80)	0.014

^a^ *N* = 33 (33 out of 38 had drop seizures).

Multivariate regression analysis again showed that complete CC (OR‐ 9.73, CI‐[1.81, 74.80], *p* = 0.014) was the only factor that significantly that increased the likelihood of complete freedom from drop seizures. None of the other variables including age at CC or duration of epilepsy affected the odds of elimination of drop seizures (*p* > 0.05 for all).

## 4 DISCUSSION

In this study, we found that complete CC was significantly more likely to lead to improvement/freedom from drop seizures when compared to anterior two‐thirds CC with increased odds of both ≥50% reduction (*p* = 0.006) as well as freedom from drop seizures (*p* = 0.014). Our study provides additional support for the higher efficacy of complete CC over anterior two‐thirds CC for treatment of drop seizures.^2^, ^3^, ^6^, ^7^, ^10^ None of the other factors examined including age at CC and duration of epilepsy correlated with drop seizure outcomes in our cohort.

Studies examining the correlation between seizure outcomes and age at CC have shown mixed results^5^, ^11^ largely due to methodological differences in outcome measures (lack of consistent definition of good outcome), patient selection (variable ages at surgery ranging from 3 to 55 years) and types of surgery performed (solely anterior CC). One study noted a trend for better seizure outcomes following CC at younger age^5^ while another failed to find an association between age at CC and seizure outcomes.^7^ Excellent seizure outcomes for epileptic spasms but not for tonic seizures have been reported for patients with WS undergoing CC (mean age of 22.6 months),^8^ perhaps suggesting a role for seizure type in determining outcomes. In our cohort, age at CC did not influence outcomes for drop seizures. Reasons for lack of influence of age at CC on seizure outcomes in our cohort are unclear. In general, younger patients tend to undergo complete CC and it is plausible that the better outcomes reported for the younger patients in other studies may reflect the effectiveness of extent of CC rather than age at CC. Our results support this hypothesis as we found that extent of CC (complete CC) was the primary predictor of treatment response after adjusting for other explanatory variables including age at surgery and duration of epilepsy.

Our study did not evaluate developmental outcomes following CC. In resective epilepsy surgery, age at surgery <12 months and shorter preoperative duration of epilepsy lead to improved developmental outcomes.^12^ Similarly, it has been reported that progressive declines in developmental quotient were prevented in patients with epileptic spasms who underwent early CC and had worthwhile improvement in seizure outcomes.^8^ Previous studies have also shown that callosotomy at an early age led to an improvement in daily function, family satisfaction and improved quality of life.^4^, ^6^ Hence, despite the lack of correlation of age at surgery or duration of epilepsy on seizure outcomes in our cohort, it is essential to identify candidates for CC early to improve outcomes.

A systematic review concluded that when compared with anterior two‐thirds CC, complete CC was significantly more likely to result in a reduction in drop seizures^2^ based on three studies reporting Engel outcomes. Contrary to the conclusions of the systematic review, a large multi‐center pediatric series failed to demonstrate the superiority of complete CC over anterior two‐thirds CC for freedom from drop attacks.^13^ However, data across the literature does suggest lower rates of seizure freedom with anterior two‐thirds CC compared to complete CC.^3^ Our study corroborates existing literature on the effectiveness of complete CC over anterior two‐thirds CC. Despite having a smaller sample size than prior studies, we demonstrated improved seizure outcomes (for drop seizures) with complete CC perhaps related to consistency of patient selection, surgical approach, and confirmation of extent of CC using intraoperative and/or postoperative MRI.

Strengths of the study include a single institutional study with homogenous patient selection, vEEG confirmation of type of drop attacks in majority and a comparatively younger cohort. A small sample size and retrospective study methodology may have potentially limited our ability to detect a clinically meaningful difference for influence of age at CC on seizure outcomes. Other limitations of the study include reliance on caregivers for quantification of seizures, limited number of patients with nondrop seizures, lack of formal neuropsychological testing, and relatively shorter duration of follow‐up.

## 5 CONCLUSION

Complete CC was significantly more likely to lead to improvement/freedom from drop seizures. Age at CC or duration of epilepsy did not influence drop seizure outcomes.

## CONFLICT OF INTEREST STATEMENT

None of the authors has any conflict of interest to disclose. We confirm that we have read the Journal's position on issues involved in ethical publication and affirm that this report is consistent with those guidelines.

## Data Availability

The datasets analyzed during the current study are available from the author on reasonable request.
